# Changes in the bony alignment of the foot after tendo-Achilles lengthening in patients with planovalgus deformity

**DOI:** 10.1186/s13018-021-02272-1

**Published:** 2021-02-08

**Authors:** Nak Tscheol Kim, Young Tae Lee, Moon Seok Park, Kyoung Min Lee, Oh Sang Kwon, Ki Hyuk Sung

**Affiliations:** 1 Department of Orthopedic Surgery, Seoul National University Bundang Hospital, 82 Gumi-ro 173 Beon-gil, Bundang-Gu, Sungnam, Gyeonggi 13620 Republic of Korea; 2 Department of Orthopedic Surgery, Seoul National University College of Medicine, Seoul, Republic of Korea

**Keywords:** Planovalgus foot deformity, Achilles tendon contracture, Tendo-Achilles lengthening, Bony alignment

## Abstract

**Background:**

This study was performed to investigate the change in the bony alignment of the foot after tendo-Achilles lengthening (TAL) and the factors that affect these changes in patients with planovalgus foot deformity.

**Methods:**

Consecutive 97 patients (150 feet; mean age 10 years; range 5.1–35.7) with Achilles tendon contracture (ATC) and planovalgus foot deformity who underwent TAL were included. All patients underwent preoperative and postoperative weight-bearing anteroposterior (AP) or lateral (LAT) foot radiographics. Changes in AP talo-1st metatarsal angle, AP talo-2nd metatarsal angle, LAT talo-1st metatarsal angle, and calcaneal pitch angle and the factors affecting such changes after TAL were analyzed using lineal mixed model.

**Results:**

There were no significant change in AP talo-1st metatarsal angle and AP talo-2nd metatarsal angle after TAL in patients with cerebral palsy (CP) (*p* = 0.236 and 0.212). However, LAT talo-1st metatarsal angle and calcaneal pitch angle were significantly improved after TAL (13.0°, *p* < 0.001 and 4.5°, *p* < 0.001). Age was significantly associated with the change in LAT talo-1st metatarsal angle after TAL (*p* = 0.028). The changes in AP talo-1st metatarsal angle, AP talo-2nd metatarsal angle, and calcaneal pitch angle after TAL were not significantly associated with the diagnosis (*p* = 0.879, 0.903, and 0.056). However, patients with CP showed more improvement in LAT talo-1st metatarsal angle (− 5.0°, *p* = 0.034) than those with idiopathic cause.

**Conclusion:**

This study showed that TAL can improve the bony alignment of the foot in patients with planovalgus and ATC. We recommend that physicians should consider this study’s findings when planning operative treatment for such patients.

## Background

Planovalgus foot deformity is one of the common foot deformities that is characterized by hindfoot valgus, decreased medial longitudinal arch, and forefoot abduction [1–5]. The cause of this deformity is unclear, and it is considered an idiopathic etiology [2, 3]. It is often associated with the Achilles tendon contracture (ATC), which causes pain and functional disability [6, 7]. It is common for patients with cerebral palsy (CP) to have planovalgus foot deformity accompanied by ATC [8].

A passive stretching exercise of the Achilles tendon with the heel in an inverted position is recommended for patients with ATC [3, 4]. Botulinum toxin injection can also be considered, especially in CP patient [9]. If patients do not respond to conservative treatment, operative treatment is indicated. Various operative methods such as arthroereisis, lateral column lengthening osteotomy, calcaneal medial displacement osteotomy, calcaneal-cuboid-cuneiform osteotomy, and arthrodesis can be considered to treat planovalgus foot deformity [3–5, 8, 10–14]. If ATC is present, tendo-Achilles lengthening (TAL) should be performed concomitantly with the planovalgus foot surgery [15–17].

Limited ankle dorsiflexion (ADF) due to ATC results in midfoot breakage and planovalgus foot deformity as ADF occurs at the mid-tarsal joint [18, 19]. Thus, we think that planovalgus foot deformity may be improved after TAL in terms of radiographic parameters as improvement in dorsiflexion at ankle joint following TAL results in the improvement of midfoot breakage. However, there has been no evidence that isolated TAL changes the degree of planovalgus foot deformity. Therefore, we performed this study to investigate the changes in bony alignment of the foot after TAL and the affecting factors in patients with planovalgus foot deformity.

## Materials and methods

This study was approved by Institutional Review Board at our hospital. The need for obtaining informed consent from the study subjects was waived due to the retrospective nature of our study.

Inclusion criteria were as follows: (1) consecutive patients who underwent TAL for ATC, (2) patients who had planovalgus foot deformity, and (3) patients who had preoperative and postoperative standing foot radiographs. Patients who underwent concomitant foot surgery for the correction of planovalgus foot deformity and those who did not have the relevant radiographs were excluded from this study. We reviewed the medical records and gathered data pertaining to patient demographics including age at surgery, sex, follow-up duration, diagnosis (cerebral palsy vs. idiopathic cause), and gross motor function classification system (GMFCS) level.

ATC was defined as ADF < 0° with knee extension during physical examination. During measurement of ADF, the foot was inverted to lock the mid-tarsal joint so as to avoid ADF at the mid-tarsal joint.

Our clinical data warehouse included 237 patients with 387 feet. After implementation of the inclusion and exclusion criteria, 97 consecutive patients (150 feet) were finally enrolled in this study. The mean age at the time of surgery was 10.0 years (range 5.1–35.7), and mean follow-up duration was 2.7 years (range 0.5–10.5). The etiology of patients was idiopathic in 8 patients (15 feet) and cerebral palsy in 89 patients (135 feet) (Table 1).

**Table 1:** Summary of patient demographics and clinical characteristics

Parameters	Values
Sex (male/female)	59/38
Age at surgery (years)	10.0 ± 5.9 (range 5.1–35.7)
Body side (right/left)	76/74
Etiology (cerebral palsy/idiopathic)	89 (135 feet)/8 (15 feet)

### Operative protocol

TAL was performed using coronal *Z*-plasty, and the intraoperative goal was to achieve ankle dorsiflexion of 10° in knee extension [20]. After surgery, all patients had to use a short leg cast and were recommended a non-weight-bearing period for 4 weeks. Thereafter, stretching, muscle-strengthening, and gait training were resumed with ankle foot orthosis, which was worn for 3 months.

### Acquisition of radiographic data

The radiographic evaluation included assessment of standing anteroposterior (AP) and lateral (LAT) foot radiographs. The foot radiographs were taken using a UT 2000 X-ray machine (Philips Research, Eindhoven, The Netherlands) at a source-to-image distance of 100 cm; the device was set to 50 kVp and 5 mAs, and radiography was performed with the patients in the standing position. The radiographic images were retrieved using a picture archiving and communication system (PACS) (IMPAX; Agfa Healthcare, Mortsel, Belgium), and radiographic measurements were performed using the PACS software.

**Fig. 1:**
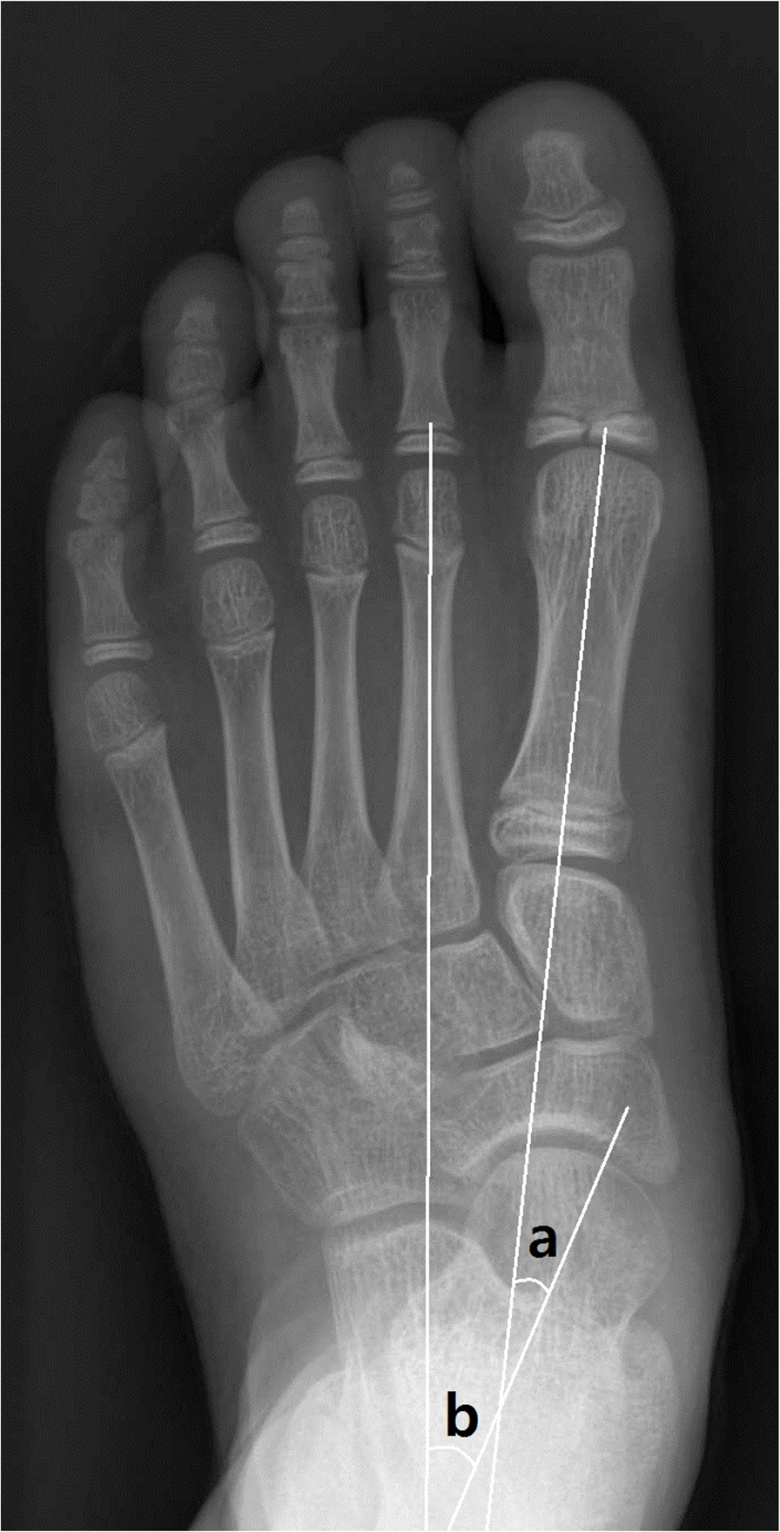
On weight-bearing anteroposterior (AP) foot radiographs, AP talo-1st metatarsal angle (**a**) and AP talo-2nd metatarsal angle (**b**) were measured

**Fig. 2:**
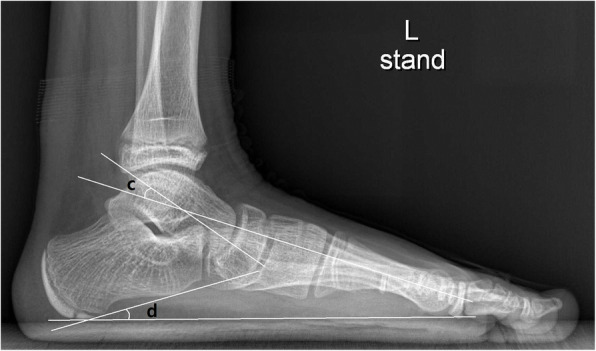
On weight-bearing lateral (LAT) foot radiographs, LAT talo-1st metatarsal angle (**c**) and calcaneal pitch angle (**d**) were measured

Four radiographic parameters on standing foot AP and LAT foot radiograph were selected to assess the degree of planovalgus foot deformity, AP talo-1st metatarsal angle, AP talo-2nd metatarsal angle, LAT talo-1st metatarsal angle, and calcaneal pitch angle [21–24]. On AP standing foot radiographs, AP talo-1st metatarsal angle was measured as the angle between a line drawn through the midpoints of the talar head and neck, and a line bisecting the long axis of the first metatarsal bone. AP talo-2nd metatarsal angle was measured as the angle between a line drawn through the midpoints of the talar head and neck, and a line bisecting the long axis of the second metatarsal bone (Fig. 1). On the LAT standing foot radiographs, LAT talo-1st metatarsal angle was measured as the angle between a line bisecting the long axis of the first metatarsal bone and a line drawn through the midpoints of the talar head and neck. Calcaneal pitch angle was measured as the angle between a line drawn through inferior border of the 5th metatarsal head and calcaneal tuberosity, and a line drawn along the lower margin of the calcaneus (Fig. 2).

Before performing the main measurements, we performed reliability testing for radiographic measurements. Sample size estimation showed that a minimum of 36 feet (18 left and 18 right) radiographs should be assessed. Three orthopedic surgeons with 3, 8, and 8 years’ experience, respectively in independent practice, measured the radiographs in a blinded fashion and determined interobserver reliability using intraclass correlation coefficients (ICCs) and 95% confidence interval.

### Statistical analyses

Reliability was assessed by the ICC in the setting of a 2-way mixed-effect model, assuming a single measurement and absolute agreement [25]. With a target ICC value of 0.8 and 95% CI width of 0.2 for 3 examiners, the minimal sample size was 36 feet using Bonett’s methods [26].

Descriptive statistics were used to summarize the patients’ demographics and radiographic measurements. Comparison between preoperative and postoperative radiographic measurements was performed using a paired *t*-test. For the purpose of statistical independence, linear mixed model (LMN) was used for statistical analysis. To estimate the extent of change of radiographic measurements after TAL, multiple factors were adjusted using a LMN with age, sex, diagnosis, and GMFCS as the fixed effects and individual subjects as the random effects.

Statistical analyses were performed using the SPSS software for Windows (version 25.0; SPSS, Inc., Chicago, IL, USA) and R version 3.5.3 (R Foundation for Statistical Computing, Vienna, Austria). All statistics were 2-tailed, and *p*-values < 0.05 were considered significant.

## Results

All radiographic measurement showed excellent interobserver reliabilities (ICC with 0.914 to 0.968). All radiographic parameters significantly improved after TAL (all *p* < 0.001) (Table 2).

**Table 2:** Changes in radiographic measurements after tendo-Achilles lengthening

Parameters (°)	Total			Cerebral palsy			Idiopathic			*p* (between group)
	Preoperative	Postoperative	*p*	Preoperative	Postoperative	*p*	Preoperative	Postoperative	*p*	
AP talus-1st metatarsal angle	14.8 ± 8.1	11.9 ± 10.5	0.017	14.8 ± 8.9	11.8 ± 11.4	0.054	14.9 ± 5.0	12.1 ± 6.7	0.045	0.190
AP talus-2nd metatarsal angle	24.5 ± 8.1	20.4 ± 11.7	0.006	24.0 ± 8.8	19.8 ± 12.7	0.026	26.1 ± 4.8	22.4 ± 7.1	0.011	0.444
Lateral talus-1st metatarsal angle	21.5 ± 10.8	12.2 ± 10.2	< 0.001	22.2 ± 10.8	12.4 ± 10.3	< 0.001	15.0 ± 8.6	10.3 ± 8.7	0.001	0.022
Calcaneal pitch angle	9.3 ± 7.0	13.3 ± 6.2	< 0.001	8.4 ± 6.4	12.6 ± 5.8	< 0.001	17.0 ± 7.4	19.2 ± 7.0	0.003	0.021

After adjusting for multiple factors, there were no significant change in AP talo-1st metatarsal angle and AP talo-2nd metatarsal angle after TAL in patients with CP (*p* = 0.236 and 0.212). However, LAT talo-1st metatarsal angle and calcaneal pitch angle were significantly improved after TAL (all *p* < 0.001). Age was significantly associated with the change in LAT talo-1st metatarsal angle after TAL (*p* = 0.028). Sex and GMFCS level were not associated with the change in radiographic parameters (Table 3).

**Table 3:** Change of radiographic parameters and affecting factors after tendo-Achilles lengthening in patients with cerebral palsy

	AP talo-1st metatarsal angle			AP talo-2nd metatarsal angle			Lateral talo-1st metatarsal angle			Calcaneal pitch angle		
	Estimate	SE	*p*-value	Estimate	SE	*p*-value	Estimate	SE	*p*-value	Estimate	SE	*p*-value
Duration	− 5.0	4.1	0.236	− 6.5	5.1	0.212	− 13.0	2.1	< 0.001	4.5	0.9	< 0.001
Sex	− 1.4	3.3	0.378	− 0.7	4.1	0.868	0.2	1.7	0.899	1.3	0.7	0.067
GMFCS level (I)												
II	0.1	3.4	0.971	1.0	4.3	0.816	1.1	1.8	0.533	− 1.1	0.8	0.173
III	− 6.6	4.8	0.174	− 7.0	5.9	0.241	− 2.6	2.3	0.253	1.3	1.0	0.180
Age	0.4	0.2	0.081	0.4	0.3	0.166	0.3	0.1	0.028	− 0.1	0.1	0.124

*SE* standard error

Change in AP talo-1st metatarsal angle, AP talo-2nd metatarsal angle, and calcaneal pitch angle after TAL were not significantly associated with the diagnosis (*p* = 0.879, 0.903, and 0.056, respectively). However, change in the LAT talo-1st metatarsal angle was significantly affected by the diagnosis (*p* = 0.034). Patients with cerebral palsy showed more improvement in LAT talo-1st metatarsal angle (− 5.0°) than those with idiopathic cause (Table 4).

**Table 4:** Change of radiographic parameters after tendo-Achilles lengthening according to diagnosis

	AP talo-1^st^ metatarsal angle			AP talo-2^nd^ metatarsal angle			Lateral talo-1^st^ metatarsal angle			Calcaneal pitch angle		
	Estimate	SE	*p*-value	Estimate	SE	*p*-value	Estimate	SE	*p*-value	Estimate	SE	*p*-value
Duration	− 3.3	3.6	0.363	− 3.5	4.5	0.441	− 7.8	2.8	0.006	2.4	1.2	0.051
Sex	− 2.1	2.5	0.421	− 2.4	3.1	0.448	0.8	1.5	0.574	0.7	0.7	0.258
Diagnosis	− 0.4	2.9	0.878	− 0.4	3.5	0.903	− 5.0	2.4	0.034	2.0	1.0	0.056
Age	0.2	0.2	0.198	0.2	0.2	0.408	0.3	0.1	0.056	-0.1	0.1	0.212

*SE* standard error

During the follow-up, calcaneal pitch angle significantly increased by 0.2° per year (*p* = 0.017). However, there were no significant changes in AP talo-1st metatarsal angle, AP talo-2nd metatarsal angle, and LAT talo-1st metatarsal angle (*p* = 0.477, 0.269, and 0.076, respectively).

## Discussion

This study was performed to investigate the extent of radiographic changes in the foot and the factors affecting such as changes after TAL in patients with planovalgus foot deformity. This study found that two LAT radiographic parameters (LAT talo-1st metatarsal angle and calcaneal pitch angle) were considerably improved after TAL, and age and diagnosis were considerably associated with these changes.

There are some limitations to this study. First, the outcomes of TAL were assessed only by the radiographic parameters, although clinical outcomes such as foot pressure distribution and questionnaire are also important after surgery. Therefore, further study including clinical outcomes after TAL is needed. Second, although radiographic measurement of the hindfoot alignment view was important for evaluating hindfoot alignment, it was not performed due to retrospective design of this study. However, it has been reported that the radiographic parameters on standing foot AP and lateral radiographs were clinically relevant in terms of reliability, discriminant validity, and convergent validity [27]. Third, the follow-up period was not long enough to evaluate the long-term effects of TAL on radiographic foot alignment although this study’s findings indicate no deterioration in terms of the radiographic parameters assessed. In order to investigate the long-term prognosis, additional long-term follow-up study is required.

If the ADF is restricted due to ATC, ADF occurs at the mid-tarsal joint, and this may eventually lead to midfoot breakage and planovalgus foot deformity [18, 19]. ATC can be treated by TAL, which enables ADF at the ankle joint. Therefore, TAL can result in the improvement of midfoot breakage and LAT radiographic parameters. LAT talo-1st metatarsal angle is the angle between the longitudinal axis of the first metatarsal bone and talus on a LAT weight-bearing foot radiograph. Increased LAT talo-1st metatarsal angle indicates a more vertically positioned talus, which represents increased midfoot breakage and increased dorsiflexion at the mid-tarsal joint. Calcaneal pitch angle represents the relative position of the calcaneus to the ground, and decreased calcaneal pitch angle means increased dorsiflexion at the mid-tarsal joint. Therefore, our result that LAT radiographic parameters were considerably improved after TAL is a reasonable finding. The degree of improvement of planovalgus foot deformity was limited despite the considerable changes in radiographic parameters after TAL. Accordingly, other operative procedure to correct planovalgus foot deformity, such as lateral column lengthening, calcaneo-stop, calcaneal-cuboid-cuneiform osteotomy, and calcaneal medial displacement osteotomy should be considered if the deformity is severe.

**Fig. 3:**
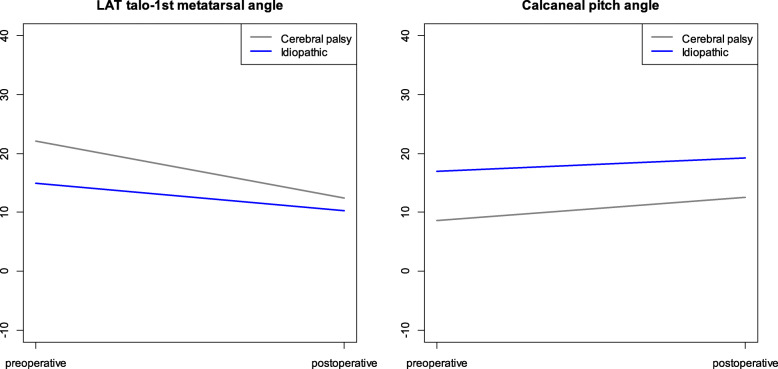
Change of LAT talo-1st metatarsal angle and calcaneal pitch angle after tendo-Achilles lengthening

In this study, the diagnosis (cerebral palsy vs. idiopathic cause) was considerably associated with the change of LAT talo-1st metatarsal angle after TAL. LAT talo-1st metatarsal angle improved more (by 5°) in patients with cerebral palsy than in those with an idiopathic deformity (Fig. 3). We think that this finding might be attributed to the following factors. First, the sample size for patients with idiopathic cause was too small in this study (8 patients, 15 feet). Therefore, further research with large number of patients with idiopathic cause is needed. Second, the degree of equinus deformity may be severe in patients with cerebral palsy than in patients with idiopathic cause. Therefore, degree of TAL might affect the change in radiographic parameters.

This study also found that age was considerably associated with the change in LAT talo-1st metatarsal angle after TAL. As the age at the time of operation increases by 1 year, the change in LAT talo-1st metatarsal angle decreases by 0.3°. Therefore, we think that it is better not to delay TAL to prevent the aggravation of planovalgus foot deformity in patients with ATC and midfoot break.

## Conclusions

In conclusion, this study showed that TAL can improve the bony alignment of the foot in patients with planovalgus foot deformity and ATC. We recommend that physicians should consider this study’s findings when planning operative intervention for such patients.

## Data Availability

The datasets used and/or analyzed during the current study available from the corresponding author on reasonable request.

## Abbreviations

ATC: Achilles tendon contracture
CP: Cerebral palsy
TAL: Tendo-Achilles lengthening
GMFCS: Gross motor function classification system
AP: Anteroposterior
LAT: Lateral
ICC: Intraclass correlation coefficient
LMN: Linear mixed model
ADF: Ankle dorsiflexion
